# P-388. A Cluster of *Pseudomonas* Infections After Vascular Surgery

**DOI:** 10.1093/ofid/ofae631.589

**Published:** 2025-01-29

**Authors:** Palak Patel, Vera Chu, Aurea Enriquez, R Marrs, Emily Landon, Patricia D Zuccaro

**Affiliations:** The University of Chicago, Chicago, Illinois; UChicago Medicine, Chicago, Illinois; University of Chicago, Chicago, Illinois; University of Chicago Medicine, Chicago, Illinois; The University of Chicago Medicine, Chicago, Illinois

## Abstract

**Background:**

Deep surgical site infection after vascular surgery is a dreaded complication. In October 2023 a vascular surgeon raised concerns about an increase in post-operative Pseudomonas infections with the infection prevention and control team at the University of Chicago Medicine.

**Methods:**

University of Chicago Medicine is an 800 bed tertiary academic medical center with an active vascular surgery practice. Investigation of the suspected cluster was conducted by the Infection Prevention and Control (IPC) team and included molecular analysis of clinical isolates, environmental cultures of shared equipment and supplies, observation of operative practices, and observation on daily bedside rounds. Available Pseudomonas strains from patients included in the investigation were sent to Mayo labs (Rochester, Minnesota) for sequencing and relatedness analysis.

**Results:**

Between 10/3/2023 and 11/3/2023, a total of 5 patients with Pseudomonas groin infection after vascular surgery were identified. 2 isolates were available for sequencing and were not related. Inspection of the vascular surgery workroom and supply room yielded shared equipment and supplies that were cultured by the IPC team. Pseudomonas was not recovered in these cultures but items from a surgical supply bag grew various gram positive organisms and a black mold that was not further identified. OR observation of operative practices revealed inadequate prep time of moist sites, lack of glove change before closing, and high traffic/occupancy during the case. Observation of daily rounds revealed multiple instances of failure to clean shared equipment (like portable ultrasound machines) between patients, lack of compliance with PPE for contact precautions, and inadequate hand hygiene practices.

**Conclusion:**

Although these cases were not clonally related, the shared organism sparked high concern on the part of the surgeon which led to swift investigation and faster remediation than would have been possible with routine surgical site infection surveillance timelines. The investigation was able to identify multiple opportunities to improve routine infection prevention practices in the care of vascular surgery patients at this hospital.

**Disclosures:**

**All Authors**: No reported disclosures

